# Sustainable Extraction of the Tetrahydropalmatine Alkaloid from *Stephania rotunda* Lour. Tubers Using Eco-Friendly Solvent Systems

**DOI:** 10.1021/acsomega.5c05568

**Published:** 2025-08-27

**Authors:** Khan Viet Nguyen, Linh Khanh Thi Nguyen, Nhan Trong Le, Duc Viet Ho, Jyrki Heinämäki, Ain Raal, Hoai Thi Nguyen

**Affiliations:** † Faculty of Pharmacy, Hue University of Medicine and Pharmacy, Hue University, Ngo Quyen 6, 49000 Hue City, Vietnam; ‡ Institute of Pharmacy, Faculty of Medicine, University of Tartu, Nooruse 1, 50411 Tartu, Estonia

## Abstract

Tetrahydropalmatine (THP) is the major bioactive alkaloid in *Stephania rotunda* Lour. (Menispermaceae) with well-known pharmacological properties, such as antiaddiction, antitumor, and neuroprotective activities. In this study, a novel ultrasonic-assisted extraction method for the sustainable extraction of the tetrahydropalmatine alkaloid from *S. rotunda* Lour. tubers was developed and optimized. The study reports also the development and validation of an HPLC-PDA analytical method intended for the quantification of THP in *S. rotunda* medicinal plant accompanied by a rapid and straightforward sample preparation procedure. A set of environmentally friendly and human-safe solvents was systematically evaluated for their extraction yield of THP, and the response surface methodology (RSM) was used for the optimization of extraction. Among the solvents studied, lactic acid showed the highest extraction yield under the optimized conditions of extraction: 90% lactic acid concentration, solvent-to-material ratio of 40 mL/g, and ultrasonic-assisted extraction at 65 °C for 10 min. With the ultrasound-enhanced extraction, the yield of THP was 1.2–1.4 times higher than that obtained with conventional solvent extraction methods. Moreover, XAD-8 resin demonstrated superior recovery performance with a THP recovery rate of 92.02 ± 0.60%. This is the first study encompassing a “green” ultrasonic-assisted solvent extraction, a validated quantification method, and resin adsorption techniques for the sustainable and efficient extraction and recovery of THP from *S. rotunda*.

## Graphical abstract

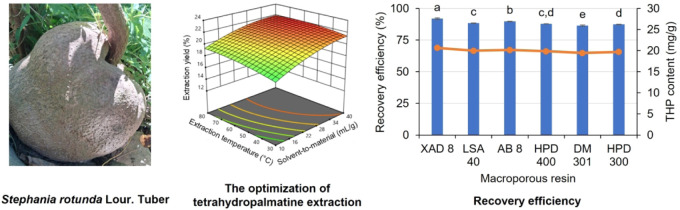

## 1 Introduction

*Stephania rotunda* Lour. is a common medicinal plant within the genus *Stephania*, which is part of the family Menispermaceae. This genus encompasses approximately 60 species, predominantly found across Southeast Asia, with around 37 species in China, 15 species in Thailand, and about 16 species in Vietnam.^1^ It has been employed in medical practice to treat various ailments, such as dysentery, fever, abdominal pain, wounds, urinary diseases, malaria, headaches, asthma, and diarrhea.^2,3^

A total of 40 alkaloids isolated from this medicinal plant (including tetrahydropalmatine (THP), cepharanthine, and xylopinine) have shown to present analgesic, anti-inflammatory, gastric contractility, renal system modulation, antiplasmodial, and acetylcholinesterase inhibitory activity.^2,4^ Tetrahydropalmatine (THP), also known as rotundine, is the principal active compound in *S. rotunda*, and it plays a key role in traditional Asian medicine. THP has attracted significant interest due to its broad spectrum of pharmacological effects, including antiaddiction, anti-inflammatory, analgesic, neuroprotective, and antitumor activity.^5,6^ In antiaddiction studies, THP showed a strong inhibitory effect on methamphetamine-induced responses in all phases of the conditioned place preference (CPP) paradigm and THP also reduced morphine-induced CPP in a dose-dependent manner.^7,8^ Moreover, THP reduced ethanol intake through the modulation of D2 receptor-mediated PKA signaling in the caudate-putamen.^9^ As an analgesic, THP alleviates acidosis-induced pain by reducing proton currents in acid-sensing ion channels (ASICs) in dorsal root ganglion neurons.^10^ It also alleviates neuropathic pain, formalin-induced pain, and bone-cancer-related pain by inhibiting microglial activation and reducing proinflammatory cytokines. Additionally, THP reduced pain in endometriosis and dysmenorrhea via oxidative stress reduction and anti-inflammatory effects.^6^ THP reduces proinflammatory cytokines such as TNF-α, IL-1β, and IL-6 and mitigates apoptosis and autophagy through pathways like TRAF6/JNK and ERK/NF-κB.^6,11^ It exhibited protective effects in disease models such as acute lung injury and myocardial ischemia-reperfusion injury, highlighting its potential for treating inflammation-related diseases.^6^ Neuroprotective effects of THP include reducing neuronal apoptosis in ischemia-reperfusion injury and oxidative stress in viral encephalitis.^12,13^ THP also modulates inflammatory pathways and neurotransmitter systems, offering potential therapeutic benefits for memory impairment, depression, and anxiety.^6^ THP has shown anticancer potential, inhibiting glioblastoma progression via suppression of the ERK/NF-κB pathway and targeting melanoma by downregulating CDK2 activity.^14,15^ In ovarian cancer, it enhances cisplatin sensitivity through the miR-93/PTEN/AKT pathway and reduces nephrotoxicity without affecting anticancer efficacy.^16,17^ Additionally, THP increases the effectiveness of tamoxifen and fulvestrant in ERα-positive breast cancer cells.^18^ Beyond cancer, THP exhibits hypotensive properties, induces vascular relaxation, inhibits osteoclastogenesis, and has antifibrotic effects. It promotes muscle regeneration, reduces liver fibrosis, and demonstrates resistance to pathogens, such as plasmodium species, parasites, and pathogenic fungi. These findings underscore THP’s potential as a multifaceted therapeutic agent.^6^ Therefore, the successful extraction and quantification of THP from medicinal plants are essential for fully exploiting its pharmacological potential.

The conventional extraction methods for THP present several limitations. These techniques often involve toxic and flammable solvents and harmful inorganic acids, thus causing true environmental and safety hazards. Moreover, they may lead to poor selectivity, low yield, and the degradation of bioactive compounds due to harsh extraction conditions.^19,20^ These drawbacks underscore the need for “greener” and more efficient extraction alternatives. The development of environmentally friendly and sustainable techniques and solvents for extracting natural products is essential to protect both the environment and public health. Additionally, such advancements play a critical role in enhancing the ecological balance, economic performance, and innovative capacity of industries.^20^

“Green” solvents stand out for their eco-friendly characteristics, such as low toxicity, thermal and chemical stability, nonflammability, and minimal volatility. These attributes position them as an attractive alternative to traditional organic solvents, particularly in light of growing environmental concerns. The researchers increasingly favor “green” solvents due to their ability to efficiently extract bioactive compounds while ensuring safety and sustainability. By reducing environmental impact and producing high-quality extracts, they address both ecological and industrial demands. The development of these solvents marks a significant leap forward in establishing safer, more sustainable extraction techniques.^21,22^

When focusing on the use of environmentally friendly (“green”) solvents and for enhancing the extraction efficiency of THP from *S. rotunda* tubers, there is also a critical need for a direct and reliable quantification method for THP in the resulting extracts. The quantification methods reported in the literature are not suitable for this purpose due to their complex sample preparation protocols, which involve multiple stages and the extensive use of organic solvents and inorganic acids.^23,24^ Previous studies on the quantification of THP utilized a detection wavelength of 282 nm.^23,24^ Although this approach proved effective, its sensitivity was limited, thus, highlighting an urgent need for the development of enhanced analytical methods to improve detection accuracy and reliability.

In the present study, we developed an eco-friendly ultrasonic-assisted extraction method for THP and explored various types of solvents to extract THP from *S. rotunda* Lour. including volatile organic compounds, inorganic acid solutions (used as controls), ionic liquids, polyalcohols, and aqueous carboxylic acid solutions. We also developed and validated a rapid HPLC-PDA method for the quantification of THP in *S. rotunda* medicinal plants accompanied by a straightforward sample preparation procedure. One key goal of the study was also to identify and find potential “green” solvents which could provide an improved extraction yield in an ultrasonic-assisted extraction over the traditional solvent-based extraction methods, while ensuring safety, cost-effectiveness, biodegradability, and regulatory approval for the use of such solvents in pharmaceutical, food, and cosmetic industries.

## 2 Materials and Methods

### 2.1 Plant Material

Tubers of *S. rotunda* were collected in Quang Tri Province, Vietnam (geographical coordinates: 16°65′93.1″N; 106°93′43.6″E). The plant material was identified by Le Tuan Anh from the Mientrung Institute for Scientific Research, Vietnam. The voucher specimens (SR-01) were deposited at the Faculty of Pharmacy, University of Medicine and Pharmacy, Hue University, Vietnam. The samples were dried, pulverized into a fine powder (1.12 kg), and passed through a 0.71 mm mesh sieve. The resulting powder was stored in a dry, light-protected environment for subsequent experimental use.

### 2.2 Standards, Reagents, and Solvents

The standard of THP was obtained from the National Institute of Drug Quality Control, Vietnam, and registered in the control logbook under code C0420141.02. A stock solution with a concentration of 1000 μg/mL was prepared by dissolving 10 mg of the THP standard in methanol (MeOH) within a 10 mL class-A volumetric flask. The calibration standards at different concentrations were subsequently obtained by diluting the stock solution with MeOH to the desired levels.

Acetone (Ace), ethanol (EtOH), MeOH, sulfuric acid (Sul), acetic acid (AA), citric acid (CA), formic acid (FA), glycolic acid (GA), lactic acid (LA), malic acid (MA), malonic acid (MLA), oxalic acid (OXA), propionic acid (PPA), pyruvic acid (PA), tartaric acid (TA), 1,2-pentanediol (C5), 1,2-hexanediol (C6), 1,2-butanediol (C4), 1,2-propanediol (PP), ethylene glycol (EG), glycerol (GL), Brij-35, Triton X-100 (TX100), Triton X-114 (TX114), Tween 40 (T40), Tween 60 (T60), Tween 65 (T65), Tween 80 (T80), and Tween 85 (T85) were purchased from Macklin Inc. (Shanghai, China). The preparation of the solvents is described in Table S1. Various resins, including DM-301, HPD-300, HPD-400, AB-8, XAD-8, and LSA-40, were provided by Tianjin Haoju Resin Technology Co., Ltd. (Tianjin, China).

### 2.3 Equipment and Instruments

The high-performance liquid chromatography (HPLC) analyses were performed using a Shimadzu LC-20A HPLC-PDA (Shimadzu Corporation, Kyoto, Japan) and an InertSustain C_18_ analytical column (5 μm, 4.6 × 250 mm^2^), protected by an InertSustain C_18_ GL-Cart guard cartridge (5 μm, 5 × 4.6 mm^2^) (MZ-Analysentechnik GmbH Barcelona, Spain). Sample preparation involved accurate weighing using a Mettler Toledo analytical balance (*d* = 0.1 mg, Mettler Toledo, Greifensee, Switzerland). UV–vis absorption spectra were recorded on a Jasco V-730 spectrophotometer (Jasco Corporation, Tokyo, Japan). Homogenization and mixing were conducted by using a Labnet VX-200 vortex shaker (Labnet International, Edison) and a Julabo SW22 thermostatic shaker (Julabo GmbH, Seelbach, Germany). Ultrasonic extraction was carried out with an Elma ultrasonic cleaner (Elma Schmidbauer GmbH, Singen, Germany), followed by sample separation through centrifugation with a Z326 K HermLe Labortechnik centrifuge (HermLe Labortechnik, Wehingen, Germany). Filtration was performed under a vacuum using a Gast DOA-P504-BN filtration system (Gast Manufacturing, Benton Harbor). Precise liquid handling was ensured using BioPette micropipettes (Eppendorf AG, Hamburg, Germany) with volumes of 10, 100, 200, and 1000 μL. Standard laboratory glassware and consumables were employed as required throughout the experimental procedures.

### 2.4 Quantification of Tetrahydropalmatine by HPLC

#### 2.4.1 Chromatographic Conditions

Quantitative analysis of all samples was conducted using reversed-phase HPLC-PDA. Chromatographic separation was achieved on an InertSustain C_18_ analytical column with a compatible guard column, as previously described. The mobile phase comprised a mixture of acetonitrile (solvent *A*) and an aqueous solution containing 0.3% phosphoric acid and 0.2% trimethylamine (solvent *B*) at a volumetric ratio of 10:90 (v/v). Analyses were performed at room temperature, employing a flow rate of 0.70 mL/min and an injection volume of 5 μL. Detection was set at 205 nm, complemented by full-spectrum scanning to ensure detailed peak characterization.

#### 2.4.2 Method Validation

The analytical method was validated in accordance with the AOAC “Guidelines for the Single Laboratory Validation of Chemical Methods for Dietary Supplements and Botanicals” to ensure its reliability and suitability for routine application. Validation was conducted prior to routine use to confirm that the method met the predefined performance criteria. The validation process included evaluation of system suitability, specificity, linearity, precision, accuracy, limit of detection (LOD), and limit of quantification (LOQ).^25^

### 2.5 Extraction of Tetrahydropalmatine from *S. rotunda*

To evaluate suitable extraction solvents and methods, 200 mg of *S. rotunda* dried tuber powder was added with 4 mL of solvents in centrifuge tubes, and the mixture was subjected to vortex agitation. The mixtures were processed in an ultrasonic bath at 50 °C for 30 min. Following this, the samples were centrifuged at 4000 rpm for 10 min to separate the solid residues. The resulting supernatants were diluted and passed through 0.45 μm membranes for filtration. HPLC analysis was performed on the prepared extracts, with each procedure repeated three times to ensure accuracy and consistency.

### 2.6 Response Surface Methodology (RSM)

To comprehensively evaluate the effects of operational parameters and their interactions on the extraction yield, response surface methodology (RSM) was applied to optimize the extraction conditions. A Box-Behnken design was constructed using Design-Expert software version 13 (Stat-Ease Inc., Minnesota) to optimize the extraction of THP from *S. rotunda* tubers. Statistical significance was determined at a p-value of less than 0.05. Four independent variables were examined: solvent concentration (*A*), solvent-to-material ratio (*B*), extraction time (C), and extraction temperature (*D*), as presented in Table 1. The experimental design comprised 29 runs, including five center point replicates to evaluate the repeatability and reliability of the model.

**Table 1:** Experimental Setup for the Extraction of Tetrahydropalmatine (THP) from *S. rotunda*

	assigned values for the variables		
variables	–1	0	+1
solvent concentration (%) (*A*)	10	50	90
solvent-to-material ratio (mL/g) (*B*)	10	25	40
extraction time (min) (*C*)	10	45	80
extraction temperature (°C) (*D*)	30	55	80

### 2.7 Recovery of THP from the Aqueous Extract

Six types of macroporous resins (HPD-300, HPD-400, DM-301, LSA-40, XAD-8, and AB-8) were employed in a solid–liquid extraction process to recover THP from the aqueous extract. Initially, the resin columns were preconditioned by sequentially treating them with absolute EtOH, 5% NaOH, and 5% HCl, followed by thorough rinsing with deionized water until a neutral pH was achieved. A 5.0 mL THP-containing solution was then passed through the prepared columns. THP adsorbed on the resin was eluted using absolute EtOH, followed by solvent evaporation to obtain a THP-enriched product. Recovery efficiency was calculated using the formula: RE_alka_ = (*w*/*w*_o_) × 100%, where “*w*” and “*w*_o_” represent the final and initial THP quantities, respectively.^26^

### 2.8 Data Processing

The data were analyzed using analysis of variance (ANOVA) conducted through SPSS software (version 26, IBM, Armonk, New York). Mean comparisons were performed using the least significant difference test with a significance level set at *p* ≤ 0.05. Optimization of experimental conditions was carried out with the assistance of Design-Expert software (version 13.0, Stat-Ease Inc., Minnesota).

## 3 Results and Discussion

### 3.1 Analytical Method Development and Validation

The chromatographic column, mobile phase composition, and flow rate were systematically adjusted by evaluating parameters, such as peak height, peak area, tailing factor, theoretical plate number, capacity factor, and resolution, to achieve optimal separation. The finalized chromatographic conditions are presented in Sections 2.3 and 2.4.1. Subsequently, the quantification method was validated following AOAC guidelines.^25^ Key validation parameters assessed included specificity, system suitability, linearity, precision, accuracy, LOD, and LOQ.

#### 3.1.1 Specificity

Specificity was assessed by injecting the blank solution, standard solution, test sample, and test sample spiked with the standard into the chromatographic system. The resulting chromatograms were analyzed and compared to confirm that the method can effectively distinguish the THP compound from the other components. The results are presented in Figure 1.

**Figure 1:**
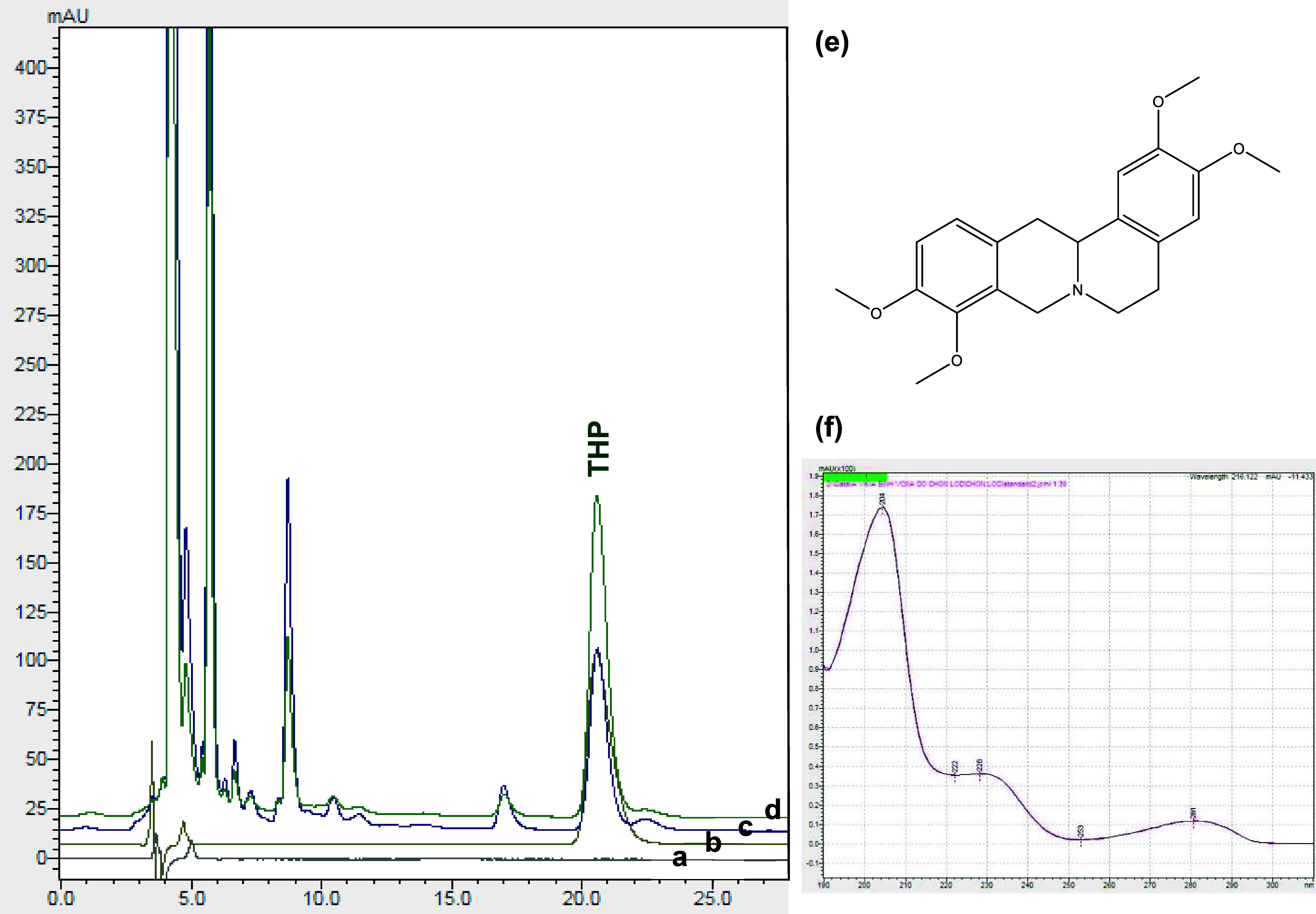
Chromatograms of the blank solution (a), standard solution (b), sample solution (c), sample solution spiked with the standard (d), the chemical structure of tetrahydropalmatine, THP (e), and superimposed UV spectra of THP in standard and sample solutions (f).

The HPLC method demonstrated high specificity as the retention time of the THP peak in the sample solution corresponded closely to that in the standard solution. No THP signal was detected in the chromatogram of the blank solution. Additionally, the UV spectrum of THP in the sample solution matched perfectly with that of THP in the standard solution.

With a higher proportion of acetonitrile (∼25 to 30%) in the mobile phase and the use of a C_18_ column, chromatograms exhibited a well-defined, sharp THP peak with a short retention time of approximately 7–10 min. The UV spectrum of this peak corresponded to that of the THP standard. However, it is noteworthy that another compound coeluted with the THP peak. According to Sothavireak,^5^ the retention time of THP was 17.386 min, showing incomplete separation from an adjacent minor peak. Under our optimized conditions, the retention time of THP was 20.691 min; an additional compound with a retention time of 22.220 min was detected, which was fully resolved from THP (Figure 1c).

#### 3.1.2 System Suitability

The standard solution at a specified concentration was injected six times consecutively. Retention time (*t*_R_), peak area (*S*_peak_), theoretical plate number (*N*), and tailing factor (*T*_f_) were recorded and evaluated. The results of the system suitability assessment are listed in Table 2.

**Table 2:** Results of the System Suitability Assessment for Tetrahydropalmatine (THP)^a^

	*t*_R_ (min)	*S*_peak_ (mAU·s)	*N*	*T*_f_
mean	20.691 ± 0.118	6094174 ± 109911	2631.978 ± 78.149	1.388 ± 0.021
RSD (%)	0.570	1.804		

a Note: *t*_R_: retention time; *S*_peak_: peak area; *N*: number of theoretical plates; *T*_f_: tailing factor.

The results of the evaluation indicated that the relative standard deviations (RSD) for the retention time and peak area were 0.570 and 1.804%, respectively, both within the acceptable limit of 2%. The tailing factor (*T*_f_) ranged between 0.8 and 1.5, while the theoretical plate number (*N*) exceeded 2000. These data confirm that the chromatographic system is suitable and adequate for the quantitative determination of the THP.

#### 3.1.3 Linearity

Linearity was evaluated by measuring the responses of standard solutions at different concentrations to examine the relationship between the signal intensity and concentration. A calibration curve was plotted based on the concentration versus peak area of quinine standards, and the correlation coefficient (*r*) was calculated.

The results, presented in Table 3, demonstrated a strong linear correlation between peak area and concentration for the THP standard over the range of 16–160 μg/mL. The calibration curve is described by the regression equation *y* = 72,735*x* – 13,758, with a coefficient of determination (*R* ^2^) of 0.9999.

**Table 3:** Linearity, Precision, and Accuracy Results of the High-Performance Liquid Chromatography (HPLC) Method for Tetrahydropalmatine (THP) Determination

parameters			results
linearity	linear range (mg/mL)		16–160
	regression equation		*y* = 72,735*x* – 13,758 (regression of *y* on *x*)
	correlation coefficient (*R* ^2^)		0.9999
precision	interday (*n* = 6)	average content of test sample (mg/g)	15.796
		RSD (%)	0.601
	intraday (*n* = 12)	average content of test sample (mg/g)	15.798
		RSD (%)	0.429
	*F* test and *t* test (*n*_1_ = 6 and *n*_2_ = 12; α = 0.05)		no statistically significant difference was observed
accuracy	no. 1	spiked amount	23.00
		recovered amount	23.34 ± 0.04
		recovery rate (%)	101.47 ± 0.21
	no. 2	spiked amount	28.75
		recovered amount	28.34 ± 0.20
		recovery rate (%)	98.59 ± 0.69
	no. 3	spiked amount	34.50
		recovered amount	35.04 ± 0.14
		recovery rate (%)	101.57 ± 0.40

#### 3.1.4 Precision

Precision was assessed by injecting a test sample (diluted 10-fold from the extract) six times on the same day under consistent analytical conditions. The procedure was repeated on a different day using the same sample to evaluate the intermediate precision. The results are summarized in Table 3.

The analysis indicated that the HPLC method demonstrated intraday repeatability with RSD values of 0.601 and 0.429% (*n* = 6), and interday precision with an RSD of 0.498% (*n* = 12). According to AOAC guidelines, for quantitative procedures with active ingredient content ≤10%, the acceptable RSD is less than 1.5%. Thus, the method complies with the AOAC precision criteria.^25^

#### 3.1.5 Accuracy

The THP standard was spiked into (10-fold-diluted) test samples to obtain three concentration levels of the analyte for recovery evaluation. Each spiked sample was injected into the HPLC system under the established analytical conditions with three replicates conducted at each concentration level. The results of the accuracy assessment are shown in Table 3. The method met the accuracy criteria, with recovery percentages ranging from 98.59% to 101.57%, in accordance with AOAC requirements.

#### 3.1.6 Limit of Detection and Limit of Quantification

Standard solutions were diluted to concentrations corresponding to signal-to-noise ratios (S/N) of 3 and 10, respectively, based on HPLC analysis results. These ratios were used to determine the LOD and LOQ, which were found to be 0.07 and 0.2 μg/mL for THP, respectively.

Overall, the developed HPLC method demonstrated acceptable system suitability and high specificity, thus ensuring accurate quantification of THP in complex sample matrices without interference from impurities. The method also exhibited a wide linear range, allowing for flexible application across various concentration levels, thereby supporting diverse analytical objectives. Evaluation of precision and accuracy parameters showed consistently high performance, confirming the method’s reliability. The analytical procedure satisfied all validation criteria recommended by AOAC guidelines and was suitable for the quantification of THP in *S. rotunda* tubers.

Based on previous studies, the determination of THP from *S. rotunda* tubers typically involves a multistep extraction procedure. In one approach, the powdered material is premoistened with hydrochloric acid–acidified water for several hours, followed by extraction with dichloromethane and subsequent evaporation to dryness. The dried residue is then reconstituted in an internal standard solution prior to the addition of the mobile phase for analysis.^23^ Alternatively, another reported method involves extraction with chloroform, evaporation to dryness using a water bath, dissolution in sulfuric acid, basification with concentrated ammonia, and re-extraction with chloroform, followed by evaporation to dryness using a water bath. The residue is then dissolved in the mobile phase and adjusted to the desired volume.^24^ These methods effectively reduce impurities and facilitate quantification of the target compound. However, they are not suitable for studies aiming to identify alternative extraction solvents (e.g., “green” solvents) to optimize the efficiency of THP extraction. The chemical composition of crude extracts analyzed directly differs from that of purified extracts obtained through extensive purification steps. Therefore, it is essential to develop a direct quantification method for THP in crude extracts that incorporates a minimal and straightforward sample preparation procedure in order to support extraction studies and related applications.

Furthermore, to the best of our knowledge, no validated analytical method has been reported for the quantification of THP in *S. rotunda* tubers using a detection wavelength of 205 nm. This highlights the importance of developing and validating such a method in the present study. Notably, the use of a detection wavelength of 205 nm—where THP exhibits significantly stronger absorbance compared to the previously reported 282 nm (as shown in Figure 1f)—resulted in substantial improvements in both the limits of detection (LOD) and quantification (LOQ). According to the study by Bory et al.,^23^ the LOD and LOQ at 282 nm were 0.75 and 2.53 μg/mL, respectively. In contrast, the values obtained in this study were approximately one-tenth of those previously reported, indicating a marked enhancement in sensitivity. This improvement is particularly advantageous for the quantification of THP in samples containing low analyte concentrations.

### 3.2 Solvent Selection for the Ultrasonic-Assisted Extraction of Tetrahydropalmatine

Inorganic acids, bases, and organic solvents were initially evaluated as the control solvents. Subsequently, eco-friendly solvents were investigated to identify the most feasible solvent for THP extraction. Standardized extraction conditions, including a solvent-to-material ratio of 20:1 (mL/g), ultrasonic extraction for 30 min, and a temperature of 50 °C, were applied to ensure consistency and comparability of results.

#### 3.2.1 Extraction Yield of Inorganic Acids, Bases, and Organic Solvents

For the extraction of THP from *S. rotunda* tubers, limewater, water, a sulfuric acid solution, and volatile organic solvents, including MeOH, EtOH, and Ace, were prepared at concentrations of approximately 100 and 50%, as detailed in Table S1. These solvents served as controls for comparative evaluation. The extraction yield of THP, presented in Figure 2 and Table S2, ranged from 2.15 to 18.06 mg/g. Remarkably, 50% MeOH yielded the highest amount of THP (18.06 mg/g), with no statistically significant difference compared to other solvents (*p* > 0.05).

**Figure 2:**
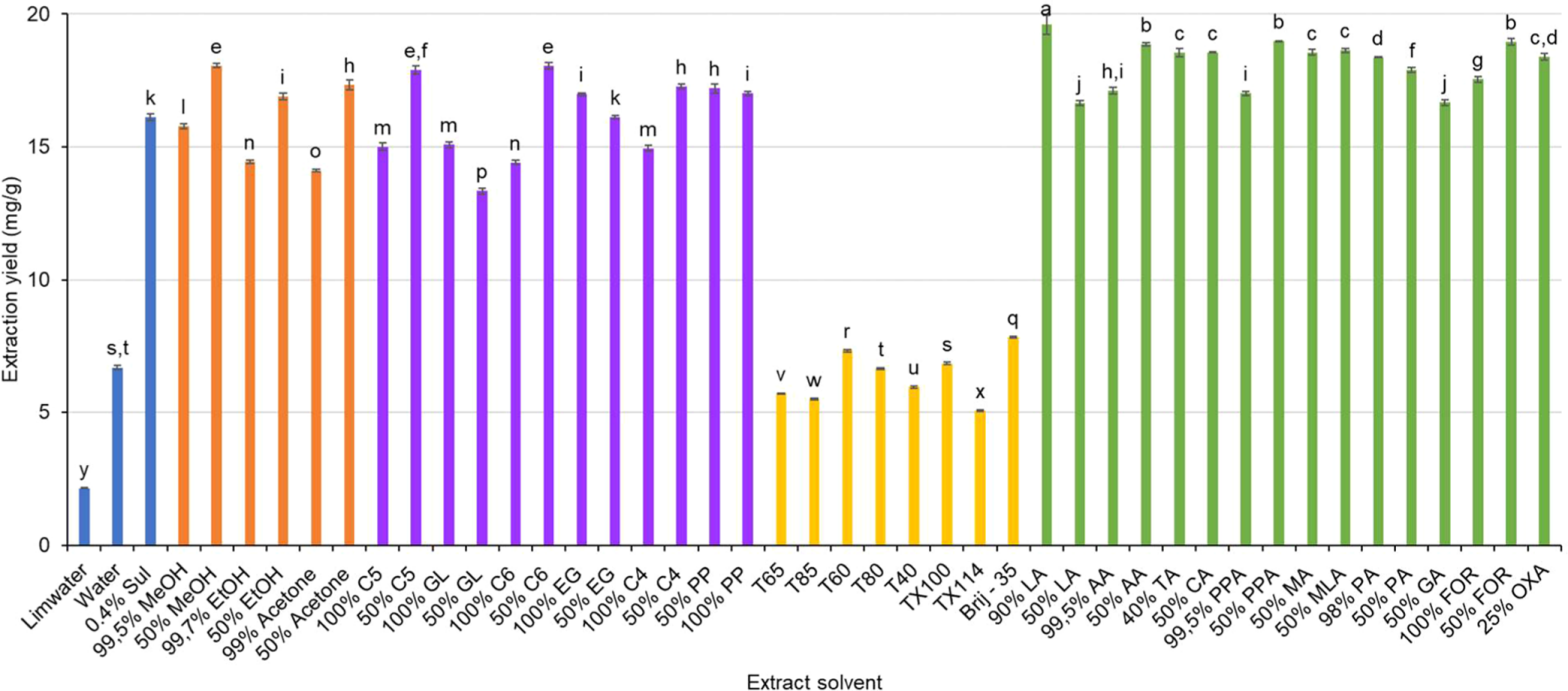
Extraction yields of tetrahydropalmatine (THP) from *S. rotunda* using solvents from the initial screening. Columns marked with different letters represent statistically significant differences (*p* < 0.05).

#### 3.2.2 Extraction Yield of Polyalcohols

To identify effective alternative solvents for extracting THP from *S. rotunda*, various polyalcohol solutions were prepared and tested (Table S1). The results presented in Figure 2 and Table S2 show that the 12 polyalcohol solutions tested for their ability to extract THP yielded the extraction values ranging from 13.34 ± 0.11 to 18.06 ± 0.12 mg/g. Among these, the 50% C6 and 50% C5 solvents exhibited the highest extraction efficiency with the yields of 18.06 ± 0.12 and 17.90 ± 0.15 mg/g, respectively (the difference between these two values was not statistically significant). However, these values did not exceed the extraction yield obtained with 50% MeOH solvent.

#### 3.2.3 Extraction Yield of Surfactant Solutions

The subsequent investigation focused on the extraction of THP using surfactants at a concentration of 5 mM. As shown in Figure 2 and Table S2, the extraction yields from the eight surfactant solutions studied were significantly lower than those obtained with organic solvents. The THP extraction yields ranged from 5.06 ± 0.03 to 7.84 ± 0.02 mg/g, and Brij-35 exhibited the highest extraction efficiency among the surfactants tested.

#### 3.2.4 Extraction Yield of Carboxylic Acid Solutions

The extraction yield of THP from *S. rotunda* using 16 carboxylic acid solutions is presented in Figure 2 and Table S2. The results show that most carboxylic acid solutions produced higher extraction yields compared to the 50% MeOH control solvent, and the THP concentrations ranged from 16.64 ± 0.09 to 19.60 ± 0.36 mg/g. Among five different solvent groups studied, including inorganic acids and bases, organic solvents, polyalcohols, surfactant solutions, and carboxylic acid solutions, the carboxylic acid group consistently exhibited a superior extraction efficiency. Markedly, the 90% lactic acid solution yielded the highest THP content (19.60 ± 0.36 mg/g).

THP is an alkaloid containing a basic amine group that readily forms salts with carboxylic acids. These salt forms exhibit high solubility in polar solvents, which enhances the extraction efficiency compared to the free base form in organic solvents. Carboxylic acids can soften or disrupt the cellular structure of *S. rotunda* tuber powder, thereby facilitating the efficient release of THP. Additionally, carboxylic acids create a mild acidic environment (typically pH 3–5), which is optimal for protonating THP without causing degradation of the active compound, as can occur under strongly acidic conditions or elevated temperatures.^27^ Compared with organic solvents, carboxylic acids allow for more selective extraction of THP, thus reducing impurities and thereby improving purification efficiency.

Among the five solvent groups studied (comprising a total of 45 solvents), 90% lactic acid solution was found to provide the highest extraction yield (Figure 2 and Table S2). The superior extraction efficiency of lactic acid compared to other carboxylic acids and solvent groups can be attributed to several factors. First, the hydroxyl (−OH) group in lactic acid increases its polarity and hydrogen-bonding capacity relative to simple carboxylic acids such as acetic, formic, and propionic acids, thus facilitating the softening of plant tissues and enhancing THP release.^28^ Second, lactic acid exhibits lower viscosity than poly­(carboxylic acid)­s like citric, malic, and tartaric acids, and therefore enabling easier penetration into plant tissues.^29^ Finally, lactic acid provides a mild pH environment that protects THP from degradation, which is superior to stronger acids such as formic, oxalic, or pyruvic acids that may cause THP decomposition.^30,31^

Noticeably, lactic acid is widely applied in the food, cosmetics, and pharmaceutical industry. In the food industry, it serves primarily as an acidulant and preservative for regulating pH and extending the product shelf life. Within the cosmetics industry, lactic acid is valued for its hydrating, antimicrobial, and skin-renewing properties, thus making it a common ingredient in skincare formulations as well as oral hygiene products. The pharmaceutical industry also benefits from lactic acid, which is incorporated into a range of medical devices and formulations, such as surgical implants, tablets, dialysis solutions, sutures, and controlled drug delivery systems. Beyond these established uses, recent advancements have expanded the role of lactic acid into the synthesis of biodegradable and biocompatible polymers like polylactic acid, and “green” solvents.^31,32^

In our current study, the 90% lactic acid solution was selected as the most feasible solvent for the further optimization of ultrasonic-assisted extraction. This choice was driven by the exceptional extraction efficiency of 90% lactic acid solution for THP. The results also suggest the potential of 90% lactic acid solution to yield even higher THP concentrations under the optimized ultrasonic-assisted extraction process conditions. Therefore, in the next step, we focused on fine-tuning the critical process parameters of the extraction to further enhance both the yield and process efficiency (reference is also made to Table 1).

### 3.3 Optimization of Ultrasonic-Assisted Extraction

For investigating the effects of process factors and their interactions on the extraction yield, we used the RSM and Box-Behnken experimental design to optimize the ultrasonic-assisted extraction process of THP from *S. rotunda* tubers.

The extraction of natural compounds from medicinal herbs involves several key stages: (1) solvent penetration into the cellular matrix of the raw material; (2) dissolution of intracellular compounds by the solvent; (3) diffusion of the dissolved compounds from the cells into the bulk extraction solvent; and (4) recovery of the extracted compounds. Enhancing the factors that promote the diffusion and solubility of the compounds during these stages can significantly improve the extraction efficiency. Critical parameters affecting the extraction performance include solvent characteristics, particle size of the raw material, solvent-to-material ratio, extraction temperature, and extraction time.^33^ In the present study, the particle size of the herbal material was standardized by sieving through a 0.71 mm mesh. The effects of the following four independent extraction process factors on the THP yield were investigated: lactic acid concentration (*A*), solvent-to-material ratio (*B*), extraction time (*C*), and extraction temperature (*D*) (Table 1). The experimental design comprised a total of 29 runs including five replicates at the center points to ensure model repeatability and reliability. The outcomes are presented in Table S3.

The results of the reliability and variance analyses are summarized in Table S4. The model demonstrated statistical significance with an *F*-value of 49.44 and a corresponding *p*-value <0.0001. The coefficient of determination (*R* ^2^ = 0.9802) alongside a lack-of-fit test value of 0.7832 (*p* > 0.05) indicated a good fit between the model and the experimental data. Furthermore, the adjusted *R* ^2^ (*R* ^2^_adj = 0.9603) and predicted *R* ^2^ (*R* ^2^_pred = 0.9200) values differ by less than 0.2, thus confirming the stability of the model and a strong predictive capability. The regression equation describing the relationship between THP extraction yield and the independent variables based on the second-order Box-Behnken RSM is presented in eq 1 (*R* ^2^ = 0.9802).

1.1 $$Y=11.15469-0.064576A+0.259528B-0.000076C+0.054389D+0.000174\mathrm{AB}-0.000385\mathrm{AC}+0.000498\mathrm{AD}+0.000548\mathrm{BC}-0.000599\mathrm{BD}+0.000014\mathrm{CD}+0.001051A^{2}-0.002942B^{2}+0.000110C^{2}-0.000587D^{2}$$

where *Y* represents the THP extraction yield (mg/g), *A* denotes the lactic acid concentration (%), *B* is the solvent-to-material ratio (mL/g), *C* corresponds to the extraction time (minutes), and *D* indicates the extraction temperature (°C).

Response surface plots visualize the interactions among independent variables and their relationship to the dependent variables. Figure 3 shows the effects of the process factors studied here on the extraction yield of THP from *S. rotunda*. Except for the interaction between temperature and extraction time, the response surfaces exhibited steep slopes, indicating a strong impact of these factors on the extraction process. These findings are supported by the analysis of variance results summarized in Table S4.

**Figure 3:**
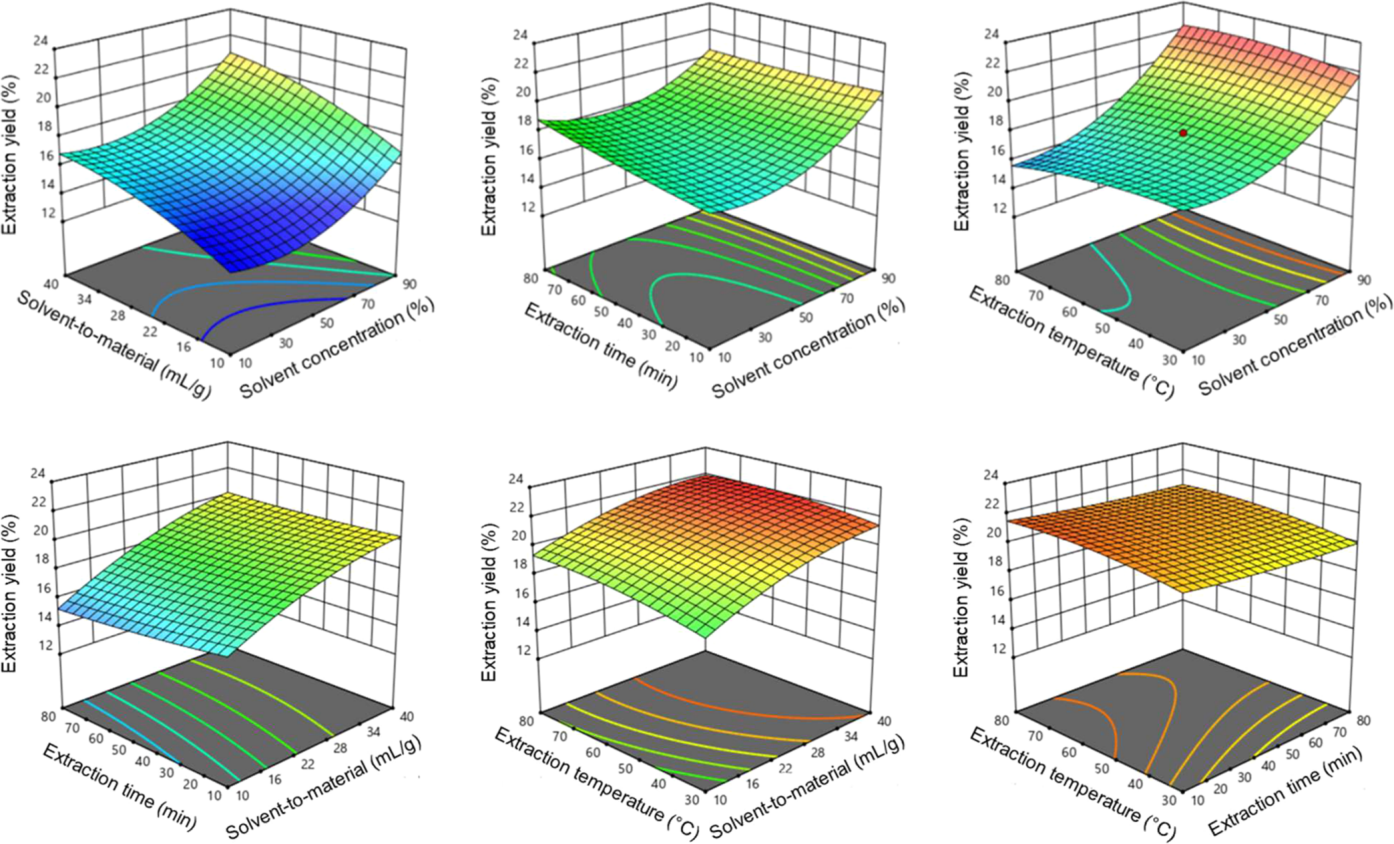
Response surface graphs illustrating the optimization of the tetrahydropalmatine (THP) extraction.

Based on the results obtained with RSM, the extraction conditions of choice were as follows: lactic acid concentration of 90.0% in the solution, solvent-to-material ratio of 39.997 mL/g, extraction time of 10.002 min, and extraction temperature of 64.004 °C. Under these conditions, the predicted THP extraction yield was 22.455 mg/g. To align with the laboratory equipment capabilities, the practical experimental conditions were slightly adjusted to lactic acid concentration of 90%, solvent-to-material ratio of 40 mL/g, extraction time of 10 min, and extraction temperature of 65 °C. Under these optimized experimental conditions, the extracted THP content was 22.450 ± 0.029 mg/g. The difference between the experimental and predicted results was not statistically significant (*p* > 0.05).

### 3.4 Recovery of Tetrahydropalmatine from *S. rotunda* Extract Using Macroporous Resins

Macroporous resins are polymeric materials characterized by highly porous structure. These materials are widely utilized for the separation and purification of bioactive compounds (including alkaloids from herbal extracts). The adsorption performance of these resins is influenced by both the physicochemical properties of the target molecules and the intrinsic structural features of the resin beads such as particle size, surface area, pore diameter, and polarity. This interaction adheres to the principle of “like dissolves like,” where polar compounds preferentially adsorb onto polar resins, while nonpolar compounds exhibit greater affinity for nonpolar resins.^34^ Adsorption mechanisms primarily involve hydrogen bonding, π-π interactions, and van der Waals forces, which are further modulated by the resin’s physical characteristics and operational parameters.^35,36^ However, excessive similarity in polarity between the macroporous resin and the target compound can lead to overly strong adsorption forces, which hinders efficient desorption and thereby have a negative impact on the recovery efficiency and reusability of the resin.^37^

In this study, six types of macroporous resins (XAD-8, LSA-40, AB-8, HPD-400, DM-301, and HPD-300) were evaluated for the recovery of THP from the *S. rotunda* extract. The results showed that the XAD-8 resin presented the highest recovery efficiency (92.02 ± 0.60%), while the DM-301 resin exhibited the lowest efficiency (86.46 ± 0.54%) (Figure 4 and Table S5). Since THP possesses moderate polarity, the moderately polar XAD-8 resin facilitates optimal van der Waals and hydrogen bonding interactions, thus leading to superior adsorption efficiency compared to nonpolar resins (such as HPD-300) and weakly polar resins (like AB-8). Although DM-301, HPD-400, and LSA-40 share similar moderate polarity, their relatively smaller pore diameters compared to XAD-8 (22.5 nm) may limit the adsorption capacity. Furthermore, the larger pore size of XAD-8 enhances desorption efficiency and minimizes the risk of pore blockage or undesired retention of adsorbates.^36^

**Figure 4:**
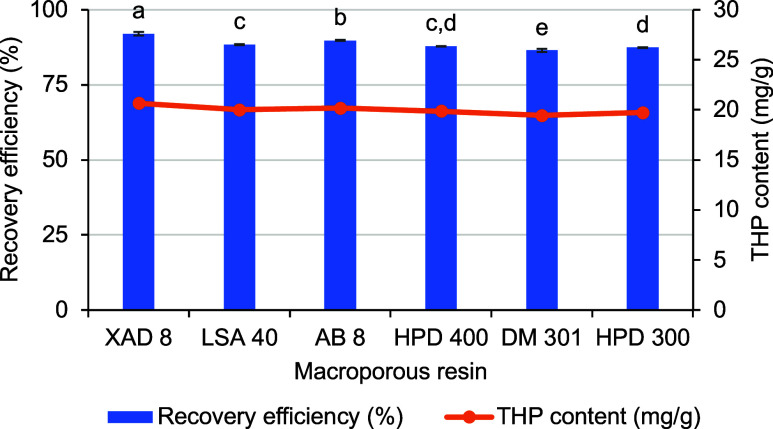
Tetrahydropalmatine (THP) recovery capacity was determined using various macroporous resins. Recovery efficiency (%) denotes the proportion of tetrahydropalmatine extracted from the solution via adsorption onto the resins. The THP content indicates the amount of tetrahydropalmatine subsequently recovered after desorption from the resin using ethanol (EtOH) as the eluent. Columns marked with different letters represent statistically significant differences (*p* < 0.05).

In general, the results of this study have significant practical implications for quality control, extraction, and related applications. Traditional extraction methods involve soaking *S. rotunda* powder in diluted H_2_SO_4_ solution for 24 h to obtain THP in its salt form, or alternatively extracting it with chloroform. The extract is subsequently dissolved in a H_2_SO_4_ solution, alkalized with ammonia or NaOH to a pH of 9–10, and subjected to chloroform extraction. This extraction step is repeated three times. The combined chloroform extracts are then concentrated by rotary evaporation and purified through crystallization in 96% EtOH.^20,38,39^ Evidently, these conventional methods consume large amounts of organic solvents and energy and require extended processing times. This study makes a practical contribution by developing an efficient ultrasonic-assisted extraction method for THP (from *S. rotunda* medicinal plant) using “green” solvents as alternatives to conventional organic solvents. The ability of ultrasonic-assisted extraction to improve alkaloid extraction efficiency compared to nonultrasonic methods has been well demonstrated. Aguilar-Hernández et al. reported that ultrasonic-assisted extraction increased the total alkaloid content in different tissues of *Annona muricata* by 2.96–56.31 times compared to maceration, with much shorter extraction times (5–15 min) depending on the matrix.^40^ Rathod and Rathod found that ultrasonic-assisted extraction yielded 5.80 mg/g piperine from *Piper longum* in only 18 min, whereas Soxhlet extraction yielded 1.67 mg/g in 4 h and batch maceration 0.98 mg/g in 8 h, corresponding to a 3.5–5.9-fold improvement.^41^ In another study, ultrasonic-assisted extraction of berberine from *Chromolaena odorata* reached 2.72% yield in 40 min, compared to only 0.22% for maceration in 60 min, representing approximately a 12.4-fold increase.^42^ Furthermore, employing a 90% lactic acid solution as the solvent system resulted in a 1.2- to 1.4-fold increase in THP yield compared to conventional solvent-based methods. Lactic acid is an environmentally friendly water-soluble material; thus, the aqueous solutions of it offer distinct advantages over potentially harmful organic solvents. The biodegradability and low toxicity of lactic acid contribute to reduced environmental impact and enhanced safety for human exposure. Lactic acid is also produced from renewable biomass, such as corn starch and sugar cane, thus decreasing reliance on fossil-based resources and mitigating greenhouse gas emissions. Moreover, it possesses strong solvent properties, enabling it to dissolve in a wide variety of media. This, in turn, broadens the utility of lactic acid in numerous industrial applications. The low vapor pressure of lactic acid reduces the level of emission of volatile organic compounds, thereby enhancing workplace safety and facilitating environmentally sustainable manufacturing processes. Taken together, these characteristics position lactic acid and its aqueous solutions as embodiments of the principles of sustainable and safe chemical practices.^30^ In the final step, the THP compound was recovered from a 90% lactic acid solution using a macroporous resin, which proved to be a convenient and efficient purification method. Conventional purification techniques, such as liquid–liquid extraction and silica gel column chromatography, present several limitations, including excessive solvent consumption, potential retention of organic solvent residues, and the generation of environmental pollutants. In contrast, macroporous resins provide notable advantages including operational simplicity, high adsorption efficiency, low operating costs, reduced solvent usage, improved product safety, and ease of regeneration. These resins have been extensively applied for the isolation and concentration of bioactive constituents from various natural sources.^35^

## 4 Conclusions

A novel ultrasonic-assisted extraction method for the sustainable extraction of the tetrahydropalmatine alkaloid from *S. rotunda* Lour. tubers was developed and optimized. In addition, a rapid and validated HPLC-PDA method using a C_18_ column (4.6 × 250 mm^2^, 5 μm) was established for quantifying THP in *S. rotunda* tubers at 205 nm. The present HPLC method requires only the dilution of the extract, followed by filtration through a 0.45 μm membrane filter, without any further sample preparation steps. The quantitative method met the AOAC validation criteria. Various eco-friendly solvents were evaluated for ultrasonic-assisted THP extraction, and 90% lactic acid solution showed the highest efficiency under optimized extraction conditions (90% lactic acid, 40 mL/g solvent-to-material ratio, 10 min extraction, 64 °C), yielding 22.450 ± 0.029 mg/g. This method improved the THP yield by 1.2 to 1.4 times compared to conventional solvents. The recovery of THP using macroporous resin XAD-8 was 92.02 ± 0.60%. To the best of our knowledge, this work presents the first comprehensive report integrating a validated HPLC-PDA quantification method, ultrasonic-assisted extraction with “green” solvents, and resin-based recovery for THP from *S. rotunda* tubers.

The improved extraction efficiency using lactic acid solvent combined with resin-based recovery methods can be applied to large-scale THP extraction, thereby promoting more sustainable and environmentally friendly practices in industrial processes.

## Supplementary Material



## Notes

Solvents used for the extraction of tetrahydropalmatine (THP) from *Stephania rotunda* (Table S1); extraction yield of tetrahydropalmatine from *Stephania rotunda* using solutions of inorganic acids, bases, organic solvents, polyalcohols, surfactants, and carboxylic acids (Table S2); extraction yields from 29 Design-Expert experimental runs (Table S3); results of analysis of variance (ANOVA) with lactic acid as the solvent (Table S4); and tetrahydropalmatine recovery capacity using various macroporous resins (Table S5) (PDF)
