# Supplementary material for: Sustainable Extraction of the Tetrahydropalmatine Alkaloid from Stephania rotunda Lour. Tubers Using Eco-Friendly Solvent Systems
Source: ACS Omega. 2025 Aug 27;10(35):40313–23. doi: 10.1021/acsomega.5c05568 (PMC12423853; doi:10.1021/acsomega.5c05568)
Supplement: Supplementary file 1 [file ao5c05568_si_001.pdf]

Supplementary material

Sustainable Extraction of Tetrahydropalmatine Alkaloid from *Stephania rotunda* Lour. Tubers Using Eco-Friendly Solvent Systems

Khan Viet Nguyen[1], Linh Khanh Thi Nguyen[1], Nhan Trong Le[1], Duc Viet Ho[1], Jyrki Heinämäki[2], Ain Raal[2,*], Hoai Thi Nguyen[1,*]

[1] Faculty of Pharmacy, Hue University of Medicine and Pharmacy, Hue University, Hue City 49000, Vietnam.

[2] Institute of Pharmacy, Faculty of Medicine, University of Tartu, 50411 Tartu, Estonia.

* Authors to whom correspondence should be addressed.

Supplementary material contents

- **Number of pages: 7**
- **Number of tables: 5**

Table S1. The solvents used as extraction solvents in the study.

Table S2. Extraction yield of tetrahydropalmatine from Stephania rotunda using solutions of inorganic acids, bases, organic solvents, polyalcohols, surfactants, and carboxylic acids.

Table S3. Extraction yields from 29 Design-Expert experimental runs.

Table S4. Results of analysis of variance (ANOVA) with lactic acid as the solvent.

Table S5. Tetrahydropalmatine recovery capacity using various macroporous resins.

Table S1. Solvents used for the extraction of tetrahydropalmatine (THP) from *Stephania rotunda*

Type	Solvent	Abbreviation	Concentration (%, w/w)
Organic solvent	Acetone	Ace	99
			50
	Ethanol	EtOH	99.7
			50
	Methanol	MeOH	99.5
			50
Inorganic solvent	Sulfuric Acid (H_2SO_4)	Sul	0.4
	Limewater	Ca(OH)2	-
	Water	H_2O	100
Carboxylic Acid	Acetic Acid	AA	99.5
			50
	Citric Acid	CA	50
	Formic Acid	FA	100
			50
	Glycolic Acid	GA	50
	Lactic Acid	LA	90
			50
	Malic Acid	MA	50
	Malonic Acid	MLA	50
	Oxalic Acid	OXA	50
	Propionic Acid	PPA	99.5
			50
	Pyruvic Acid	PA	98
			50
	Tartaric Acid	TA	50
Polyalcohol	1,2-Pentanediol	C5	100
			50
	1,2-Hexanediol	C6	100
			50
	1,2-Butanediol	C4	100

			50
	1,2-Propandiol	PP	100
			50
	Ethylene glycol	EG	100
			50
	Glycerol	GL	100
			50
Surfactant	Brij 35	Brij 35	5 mM
	Triton X100	TX100	5 mM
	Triton X114	TX114	5 mM
	Tween 40	T40	5 mM
	Tween 60	T60	5 mM
	Tween 65	T65	5 mM
	Tween 80	T80	5 mM
	Tween 85	T85	5 mM

Note: Tartaric Acid, Citric Acid, Malic Acid, Malonic Acid, Glycolic Acid, and Oxalic Acid exist in solid form, so their studied concentration is 50%.

Table S2. Extraction yield of tetrahydropalmatine from *Stephania rotunda* using solutions of inorganic acids, bases, organic solvents, polyalcohols, surfactants, and carboxylic acids

No.	Extraction solvent	Extraction efficiency(mg/g)
1	Limwater	2.15 ± 0.02^{y}
2	Water	$6.70 \pm 0.07^{s,t}$
3	H_2SO_4 0.4%	16.12 ± 0.13^{k}
4	MeOH 99.5%	15.78 ± 0.08^{l}
5	MeOH 50%	18.06 ± 0.08^{e}
6	EtOH 99.7%	14.43 ± 0.06^{n}
7	EtOH 50%	16.90 ± 0.13^{i}
8	Acetone 99%	14.11 ± 0.04^{o}
9	Acetone 50%	17.34 ± 0.19^{h}
10	C5 100%	15.01 ± 0.14^{m}
11	C5 50%	$17.90 \pm 0.15^{e,f}$
12	GL 100%	15.09 ± 0.11^{m}
13	GL 50%	13.34 ± 0.11^{p}

14	C6 100%	14.42 ± 0.08^{n}
15	C6 50%	18.06 ± 0.12^{e}
16	EG 100%	16.99 ± 0.05^{i}
17	EG 50%	16.11 ± 0.06^{k}
18	C4 100%	14.94 ± 0.11^{m}
19	C4 50%	17.27 ± 0.10^{h}
20	PP 50%	17.20 ± 0.16^{h}
21	PP 100%	17.02 ± 0.08^{i}
22	T65	5.71 ± 0.02^{v}
23	T85	5.50 ± 0.03^{w}
24	T60	7.33 ± 0.05^{r}
25	T80	6.66 ± 0.03^{t}
26	T40	5.96 ± 0.04^{u}
27	TX100	6.85 ± 0.05^{s}
28	TX114	5.06 ± 0.03^{x}
29	Brij – 35	7.84 ± 0.02^{q}
30	LA 90%	19.60 ± 0.36^{a}
31	LA 50%	16.64 ± 0.09^{j}
32	AA 99.5%	17.12 ± 0.12^{h,i}
33	AA 50%	18.86 ± 0.07^{b}
34	TA 40%	18.55 ± 0.17^{c}
35	CA 50%	18.56 ± 0.02^{c}
36	PPA 99.5%	17.02 ± 0.08^{i}
37	PPA 50%	18.99 ± 0.01^{b}
38	MA 50%	18.56 ± 0.11^{c}
39	MLA 50%	18.63 ± 0.08^{c}
40	PA 98%	18.37 ± 0.02^{d}
41	PA 50%	17.89 ± 0.10^{f}
42	GA 50%	16.68 ± 0.11^{j}
43	FOR 100%	17.54 ± 0.12^{g}
44	FOR 50%	18.95 ± 0.13^{b}
45	OXA 25%	18.39 ± 0.12^{c,d}

Columns marked with different letters represent statistically significant differences ($p < 0.05$).

Table S3. Extraction yields from 29 Design-Expert experimental runs

No.	A	B	C	D	Average yield ± SD
1	50	25	45	55	16.774 ± 0.150
2	50	25	10	80	16.811 ± 0.098
3	10	25	80	55	17.958 ± 0.227
4	50	10	45	30	14.687 ± 0.131
5	50	40	80	55	18.700 ± 0.031
6	90	25	10	55	21.499 ± 0.151
7	90	25	80	55	21.244 ± 0.081
8	90	25	45	30	19.777 ± 0.179
9	90	25	45	80	21.325 ± 0.089
10	50	10	10	55	14.835 ± 0.164
11	50	40	45	30	18.295 ± 0.155
12	50	25	10	30	17.041 ± 0.204
13	90	10	45	55	18.593 ± 0.143
14	10	25	45	30	16.313 ± 0.079
15	50	40	10	55	17.918 ± 0.163
16	50	25	45	55	17.589 ± 0.059
17	90	40	45	55	22.138 ± 0.105
18	10	25	10	55	16.055 ± 0.158
19	10	10	45	55	14.452 ± 0.073
20	50	25	45	55	17.456 ± 0.068
21	50	25	80	30	17.043 ± 0.159
22	50	40	45	80	17.552 ± 0.046
23	50	10	45	80	14.843 ± 0.051
24	50	25	45	55	16.515 ± 0.050
25	50	25	80	80	16.863 ± 0.021
26	50	10	80	55	14.467 ± 0.009
27	10	40	45	55	17.581 ± 0.036
28	50	25	45	55	17.585 ± 0.006
29	10	25	45	80	15.870 ± 0.034

Table S4. Results of analysis of variance (ANOVA) with lactic acid as the solvent

Source	Sum of Squares	df	Mean Square	F-value	p-value	
Model	122.05	14	8.72	49.44	< 0.0001	significant
A	57.84	1	57.84	328.01	< 0.0001	
B	34.36	1	34.36	194.86	< 0.0001	
C	0.3731	1	0.3731	2.12	0.1678	
D	0.0010	1	0.0010	0.0055	0.9421	
AB	0.0434	1	0.0434	0.2462	0.6275	
AC	1.16	1	1.16	6.60	0.0222	
AD	0.9911	1	0.9911	5.62	0.0326	
BC	0.3309	1	0.3309	1.88	0.1923	
BD	0.2019	1	0.2019	1.14	0.3027	
CD	0.0006	1	0.0006	0.0036	0.9532	
A^2	18.34	1	18.34	103.99	< 0.0001	
B^2	2.84	1	2.84	16.12	0.0013	
C^2	0.1177	1	0.1177	0.6677	0.4275	
D^2	0.8735	1	0.8735	4.95	0.0430	
Residual	2.47	14	0.1763			
Lack of Fit	1.45	10	0.1455	0.5737	0.7832	not significant
Pure Error	1.01	4	0.2535			
Cor Total	124.52	28				
	$R^2 = 0.9802$		R^2 adj = 0.9603		R^2 pred = 0.9200	

Table S5. Tetrahydropalmatine recovery capacity using various macroporous resins

No.	Resin name	Recovery efficiency (%)	Recovered content (mg/g)
1	XAD-8	92.02 ± 0.60^{a}	20.66 ± 0.13^{a}
2	LSA-40	88.39 ± 0.17^{c}	19.99 ± 0.27^{c}
3	AB-8	89.81 ± 0.17^{b}	20.16 ± 0.04^{b}
4	HPD 400	$87.83 \pm 0.09^{c,d}$	$19.88 \pm 0.13^{c,d}$
5	DM 301	86.46 ± 0.54^{e}	19.45 ± 0.20^{e}
6	HPD 300	87.40 ± 0.13^{d}	19.73 ± 0.21^{d}

Columns marked with different letters represent statistically significant differences ($p < 0.05$).
